# Laser‐Induced Ultrafast Magnetic Phase Transition in 2D Van Der Waals Antiferromagnetic Heterostructures

**DOI:** 10.1002/advs.202515533

**Published:** 2026-01-05

**Authors:** Yang Wu, Fulu Zheng, San‐Dong Guo, Thomas Frauenheim, Zhaobo Zhou, Junjie He

**Affiliations:** ^1^ Bremen Center for Computational Materials Science University of Bremen 28359 Bremen Germany; ^2^ School of Physical Science and Technology Ningbo University Ningbo 315211 China; ^3^ School of Electronic Engineering Xi'an University of Posts and Telecommunications Xi'an 710121 China; ^4^ School of Science Constructor University 28759 Bremen Germany; ^5^ Department of Physical and Macromolecular Chemistry Faculty of Science Charles University in Prague Prague 12843 Czech Republic; ^6^ Institute of Advanced Study Chengdu University Chengdu 610100 China

**Keywords:** 2D van der waals heterostructures, magnetic phase transition, real‐time density functional theory, spin relaxation, ultrafast spin dynamics

## Abstract

Light offers the fastest route to controlling magnetization, yet the microscopic mechanisms in 2D magnets have been less explored. Here, using real‐time time‐dependent density functional theory combined with ab initio nonadiabatic molecular dynamics, ultrafast laser‐induced spin transfer and spin relaxation dynamics in 2D van der Waals antiferromagnetic CrI_3_/CrGeTe_3_ heterostructures are investigated. Laser excitation is found to induce pronounced interlayer nonequilibrium spin dynamics and ultrafast magnetic moment reconstruction, driving a transition from an antiferromagnetic (AFM) state to a ferrimagnetic (FiM) state. This transition is governed by two key factors: i) asymmetric ultrafast demagnetization of magnetic atoms in the two ferromagnetic layers, leading to substantial changes in interlayer exchange interactions, and ii) a compensatory contribution from the magnetic moments of nonmagnetic atoms, which enhances the stability of the FiM state. Moreover, spin relaxation analysis reveals distinct relaxation mechanisms in the CrI_3_ and CrGeTe_3_ layers, where asymmetric interlayer relaxation dynamics significantly extend the FiM lifetime. These findings provide a comprehensive physical picture of laser‐induced magnetization transitions in 2D magnets, offering critical theoretical insights for the development of light‐controlled magnetic storage and ultrafast spintronic switches.

## Graphical abstract

In this work, ultrafast laser‐induced spin transfer and spin relaxation dynamics in 2D van der Waals antiferromagnetic CrI_3_/CrGeTe_3_ heterostructures are investigated. Laser excitation is demonstrated to induce pronounced interlayer nonequilibrium spin dynamics and ultrafast magnetic moment reconstruction, driving a transition from an antiferromagnetic (AFM) state to a ferrimagnetic (FiM) state.

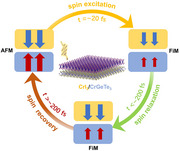

## 1 Introduction

Laser‐induced ultrafast magnetic phase transition is an important research direction in spintronics, which is capable of changing the magnetic state of materials in a very short time, thus enhancing the speed of information storage and processing. ^[^ ^1^, ^2^, ^3^, ^4^, ^5^ ^]^ However, conventional magnetic modulation methods (e.g., applied magnetic fields, gate voltages, and stress engineering) are usually slow in response and high in energy consumption.^[^ ^6^, ^7^, ^8^, ^9^ ^]^ In contrast, ultrafast laser pulses can drive magnetic order rearrangements on femtosecond (fs) and even attosecond (as) scales, enabling high‐speed magnetic switching and spin modulation.^[^ ^10^, ^11^, ^12^, ^13^ ^]^ Therefore, the study of laser control of the magnetic phase transition and the elucidation of the fundamental physical laws of the process in magnetic materials are important for the future development of ultrafast spintronic devices.

With the advent of the post‐Moore era, the discovery of intrinsic 2D magnetic materials, such as CrI_3_,^[^ ^14^ ^]^ Cr_2_Ge_2_Te_6_,^[^ ^15^ ^]^ and Fe_3_GeTe_2_,^[^ ^16^ ^]^ has reinvigorated interest in low‐dimensional magnetism, offering new opportunities for miniaturized spintronic devices. Among various 2D magnetic materials, antiferromagnetic (AFM) materials have attracted significant attention due to their intrinsic advantages over ferromagnetic (FM) counterparts, such as the absence of stray fields, robustness against external perturbations, and terahertz‐scale spin dynamics, making them promising candidates for spintronic and multiferroic heterostructures.^[^ ^17^, ^18^, ^19^ ^]^ The ultrafast response of AFM materials makes them highly suitable for next‐generation spintronic applications, including terahertz spintronic oscillators and nonvolatile memory devices.^[^ ^20^, ^21^, ^22^ ^]^ However, a major challenge in utilizing AFM materials is the intrinsic absence of a net magnetization and the often nanoscale size of magnetic domains, which makes direct control and detection difficult.^[^ ^23^, ^24^, ^25^ ^]^

To overcome this limitation, we propose an alternative to construct a 2D interlayer AFM van der Waals (vdW) heterostructure in which a CrI_3_ monolayer forms an interface with a CrGeTe_3_ layer. This heterostructure breaks the intrinsic spatial inversion symmetry of the AFM system, leading to unique interfacial interactions and band structures that may facilitate laser‐induced asymmetric interlayer demagnetization, thereby enabling an antiferromagnetic to ferrimagnetic phase transition. Compared with conventional AFM materials, this heterostructure not only enhances effective spin regulation but also realizes non‐contact, ultrafast, and highly efficient magnetic phase transitions that break through the limitations of magnetic and electric field manipulation. In addition, vdW interface engineering provides a versatile platform to modulate exchange interactions and spin transport properties on the atomic scale.^[^ ^26^, ^27^, ^28^ ^]^

In this work, we investigate the laser‐induced magnetic phase transition in an interlayer AFM vdW heterostructure, demonstrating a transition from the AFM state to the FiM state under ultrafast laser excitation. By analyzing the time evolution of atomic spin moments and electronic occupations, we reveal the underlying physical mechanisms driving this transition. Our results indicate that the differential demagnetization rates of the layers lead to a transient imbalance in the interlayer spin alignment, ultimately stabilizing the FiM state. Additionally, through ab initio nonadiabatic molecular dynamics (NAMD) simulations, we explore the spin relaxation dynamics and identify key pathways for spin angular momentum transfer. Our findings provide critical insights into ultrafast spin‐state transitions in AFM vdW systems and offer new strategies for controlling magnetic phase transitions at ultrafast timescales.

## 2 Results and Discussion

### 2.1 Ground States Structural, Magnetic, and Electronic Properties

**Figure 1:**
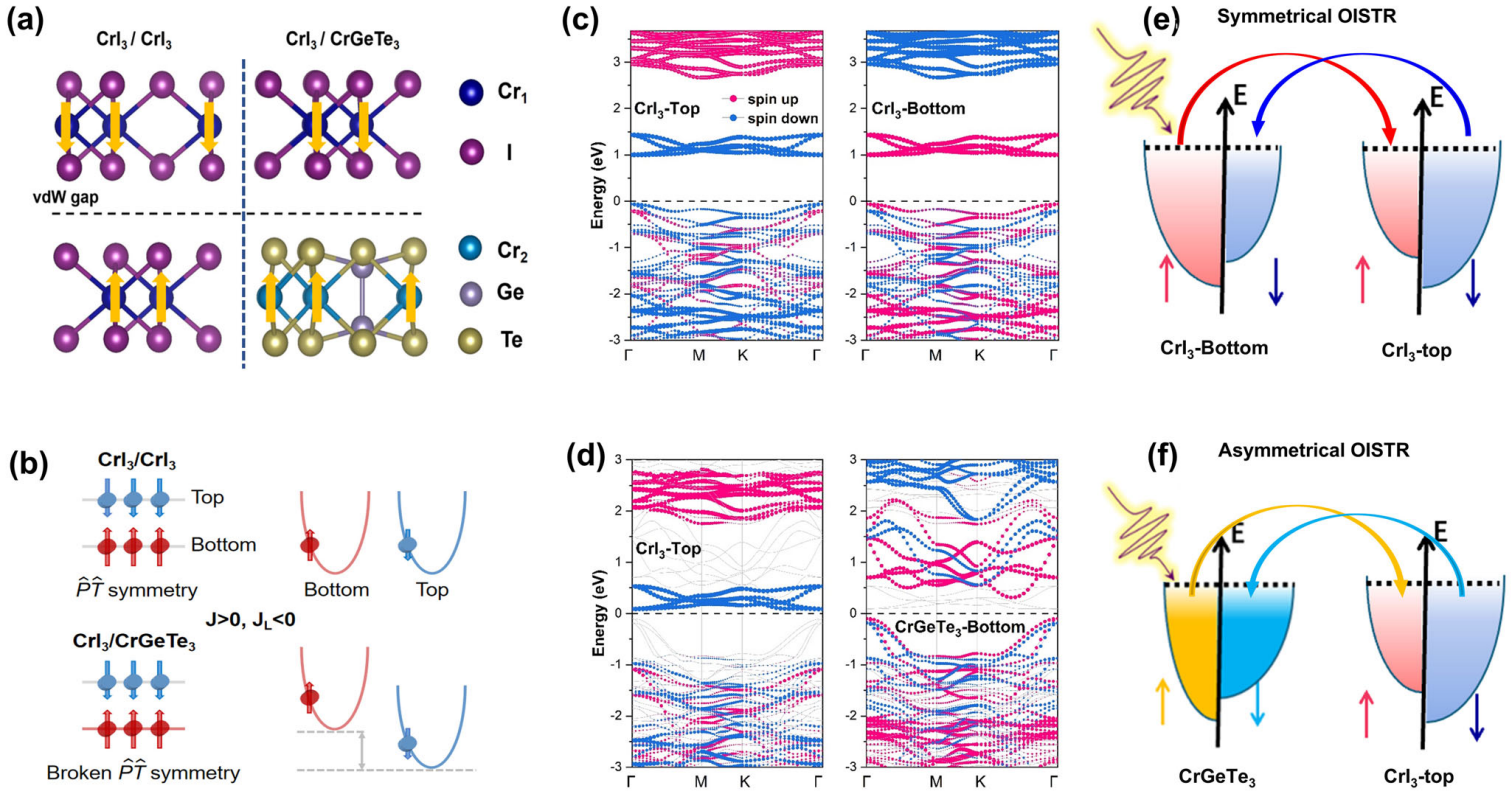
a) Spin configurations of the AB‐stacked bilayer CrI_3_ (left) and CrI_3_/CrGeTe_3_ heterostructures (right). The yellow arrows represent the direction of the magnetic order of Cr atoms. b) Schematic of the low‐energy band structure. The red (blue) bands show the dispersion relation of spin‐up (spin‐down) states in the bottom (top) layer. c,d) Layer‐projected band structures of the AB‐stacked bilayer CrI_3_ and CrI_3_/CrGeTe_3_ heterostructures. e,f) Schematic illustration of spin transfer processes in two systems.

In 2D magnetic materials, the interlayer exchange interactions play a key role in determining the magnetic ground state. For the typical AB‐stacked CrI_3_ (**Figure** **1**a‐left), the interlayer AFM arrangement is the lowest energy ground state, which has been widely verified in experimental and theoretical calculations.^[^ ^14^, ^29^ ^]^ However, the space‐time (*P̂T̂*) symmetry leads to spin degeneracy in the electronic band structure (Figure 1c; Figure S3a, Supporting Information), which suppresses the formation of spin‐polarized electronic states. To introduce tunable spin polarization, we designed and constructed a CrI_3_/CrGeTe_3_ van der Waals heterostructure. The lattice constants of CrI_3_ and CrGeTe_3_ are closely matched at 7.086 and 6.916 Å, respectively, resulting in a minimal lattice mismatch of 2.46%. Therefore, the heterostructure can be constructed directly from the unit cells. Various stacking arrangements were examined (Figure S1, Supporting Information), with binding energy calculations indicating that type B is the most energetically favorable configuration, as shown in Table S1 (Supporting Information). In the CrI_3_/CrGeTe_3_ vdW heterostructures, the coercivities of different ferromagnetic layers vary due to differences in their magnetic anisotropy energy. Therefore, two distinct spin configurations are investigated: the antiparallel (AP) arrangement, representing the interlayer AFM state, and the parallel (P) arrangement, corresponding to the FM state (Figure S2, Supporting Information). The binding energies of the CrI_3_/CrGeTe_3_ vdW heterostructures can indicate that the AFM ground state is more stable (Figure 1a‐right); therefore, our study focuses on the AP system. Furthermore, spin‐polarized calculations inform that the orbital hybridization between the magnetic Cr atoms and the nonmagnetic ligands induces local magnetic moments of 0.13/0.03/−0.15 *µ_B_* on the I/Ge/Te atoms, respectively.

In the CrI_3_/CrGeTe_3_ heterostructure, the band structures reveal that the global valence band maximum (VBM) primarily originates from the CrGeTe_3_ sublayer, while the conduction band minimum (CBM) is mainly contributed by the CrI_3_ sublayer, indicating a type‐II band alignment (Figure 1d). Furthermore, the spin‐up and spin‐down electronic states exhibit distinct spin splitting near the Fermi level. Due to the presence of spin‐polarized energy bands, the heterostructure can support the formation of nonequilibrium electronic states, which may trigger different ultrafast magnetic responses under laser excitation (Figure 1e,f).

### 2.2 Laser‐Induced Interlayer Spin Transfer Dynamics

**Figure 2:**
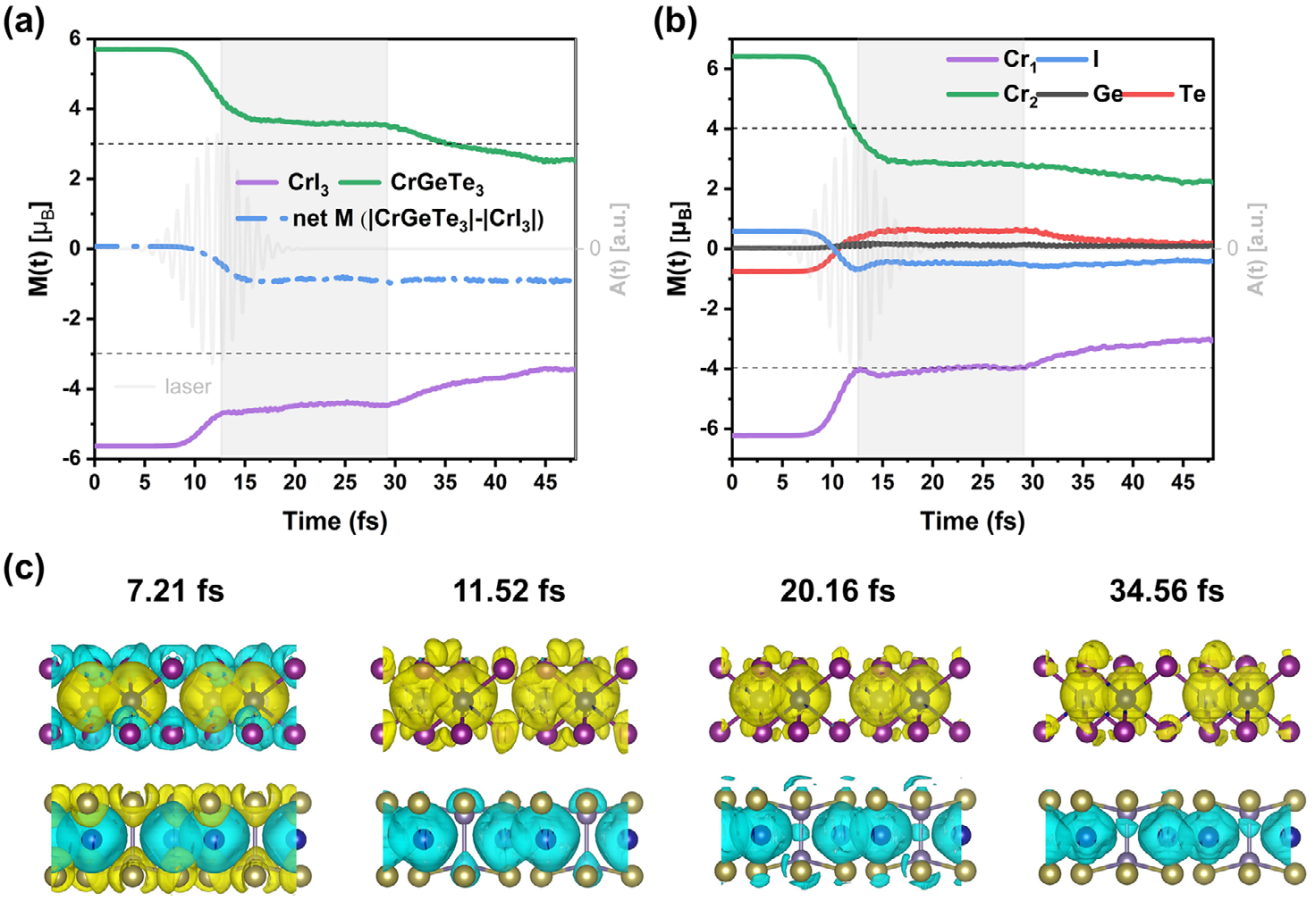
Time‐dependent spin dynamics and spin density of the CrI_3_/CrGeTe_3_ heterostructure. a) Time‐dependent dynamics of the layer magnetic moments for CrI_3_ (violet) and CrGeTe_3_ (green). b) Time evolution of the local magnetic moment, along with the vector potential A(t) corresponding to the laser pulse. c) Snapshots of the spin density at t = 7.21, 11.52, 20.16, and 34.56 fs. The isosurface is set to 0.003 e/Å^3^. Blue (yellow) indicates the spin up (down) density, respectively.

We excited the CrI_3_/CrGeTe_3_ heterostructure using a linearly polarized (in‐plane polarization) ultrafast laser pulse based on rt‐TDDFT calculations. The laser is incident normal to the heterostructure plane (along the z direction). The polarization of the laser is in‐plane, with the electric field (E‐vector) oriented along the kx direction (polarization angle θ = 0°). The laser pulse is characterized by a photon energy of 4.08 eV, a full width at half maximum of 6.04 fs, and a fluence of 13.97 mJ/cm^2^. (for the computational details, refer to the Computational Methods). The rt‐TDDFT results reveal that both CrI_3_ and CrGeTe_3_ layers showed significant ultrafast demagnetization behavior during laser excitation, as shown in **Figure** **2**a. The two layers almost synchronously undergo magnetic moment decay, but there are obvious differences in the demagnetization amplitude and time evolution. Among them, compared to CrGeTe_3_, the CrI_3_ layer exhibits a slower demagnetization response and smaller overall amplitude. This asymmetric magnetic moment evolution leads to a change of the net magnetic moment within a 9‐15 fs time window, which prompts the transition of the system from the AFM state to the FiM state. This process can be understood as a spin dynamic modulation of laser‐induced exchange interactions, which disrupts the interlayer spin alignment, leads to the generation of FiM states. Furthermore, compared with the individual CrI_3_ and CrGeTe_3_ monolayer (Figure S4, Supporting Information), the heterostructure exhibits a larger amplitude asymmetry and a unique temporal evolution in the demagnetization process. These differences go beyond a simple superposition of the intrinsic layer responses and mainly originate from the interlayer hybridization and exchange coupling at the interface.

To further understand the ultrafast demagnetization process and the laser‐induced FiM state in the CrI_3_/CrGeTe_3_ heterostructure, the time‐dependent element‐resolved spin dynamics are presented in Figure 2b. To present our data clearly, we compared the change in the magnetic moment, ∆M═M (t)‐M (t═0), as shown in Figure S6 (Supporting Information). i) During the laser excitation, the magnetic Cr_1_ atoms in the CrI_3_ layer undergo demagnetization of ≈2 *µ_B_* at the peak of the laser pulse (12 fs), whereas the Cr_2_ atoms in the CrGeTe_3_ layer exhibit stronger demagnetization effects (≈3 *µ_B_*) and their demagnetization lasts longer. This suggests that there is a significant difference in the response of magnetic Cr atoms in each layer to laser excitation, which is a key factor in the formation of interlayer FiM states. In addition, the magnetic switching of the nonmagnetic atoms I and Te further confirms the role of the optical intersite spin transfer (OISTR) effect,^[^ ^30^ ^]^ i.e., the laser excitation‐triggered transfer of spin‐polarized electrons within the layers, which affects the magnetic moment evolution within the layers. ii) Within the time window of ^[^ ^12^, ^28^ ^]^ fs, the I atoms have a significant transfer of the magnetic moment, which leads to magnetic moment compensation of Cr1 atoms, explaining why the magnetic moment of Cr1 atoms increases in this time range. However, a similar compensation effect is not observed in the CrGeTe_3_ layer, which is due to the asymmetry of the interlayer exchange interactions, leading to a different spin‐scattering mechanism. iii) After 28 fs, the nonmagnetic Te atoms exhibit pronounced demagnetization, after which the magnetic moment stabilizes at zero. However, when the magnetic moment of the Te atoms reaches zero, the demagnetization of Cr atoms in each layer continues to occur. This behavior differs from the magnetic moment compensation of I for the Cr1 atoms. It is attributed to interlayer spin transfer between CrI_3_ and CrGeTe_3_. Overall, the laser‐induced asymmetric demagnetization behavior of magnetic Cr atoms in different layers drives the transition of the interlayer magnetic moments from the AFM state to the FiM state. Meanwhile, the magnetic moment flip of the nonmagnetic I/Te atoms further enhances the stability of the FiM state. In order to further visualize the ultrafast spin dynamics process and non‐equilibrium properties in the CrI_3_/CrGeTe_3_ heterostructures, we calculated the time‐dependent spin density dynamics (Figure 2c). It clearly shows that the asymmetric demagnetization behavior of the CrI_3_ and CrGeTe_3_ layers, combined with the OISTR effect of nonmagnetic atoms I and Te, drives the transition of the interlayer magnetic moments from the AFM state to the FiM state.

**Figure 3:**
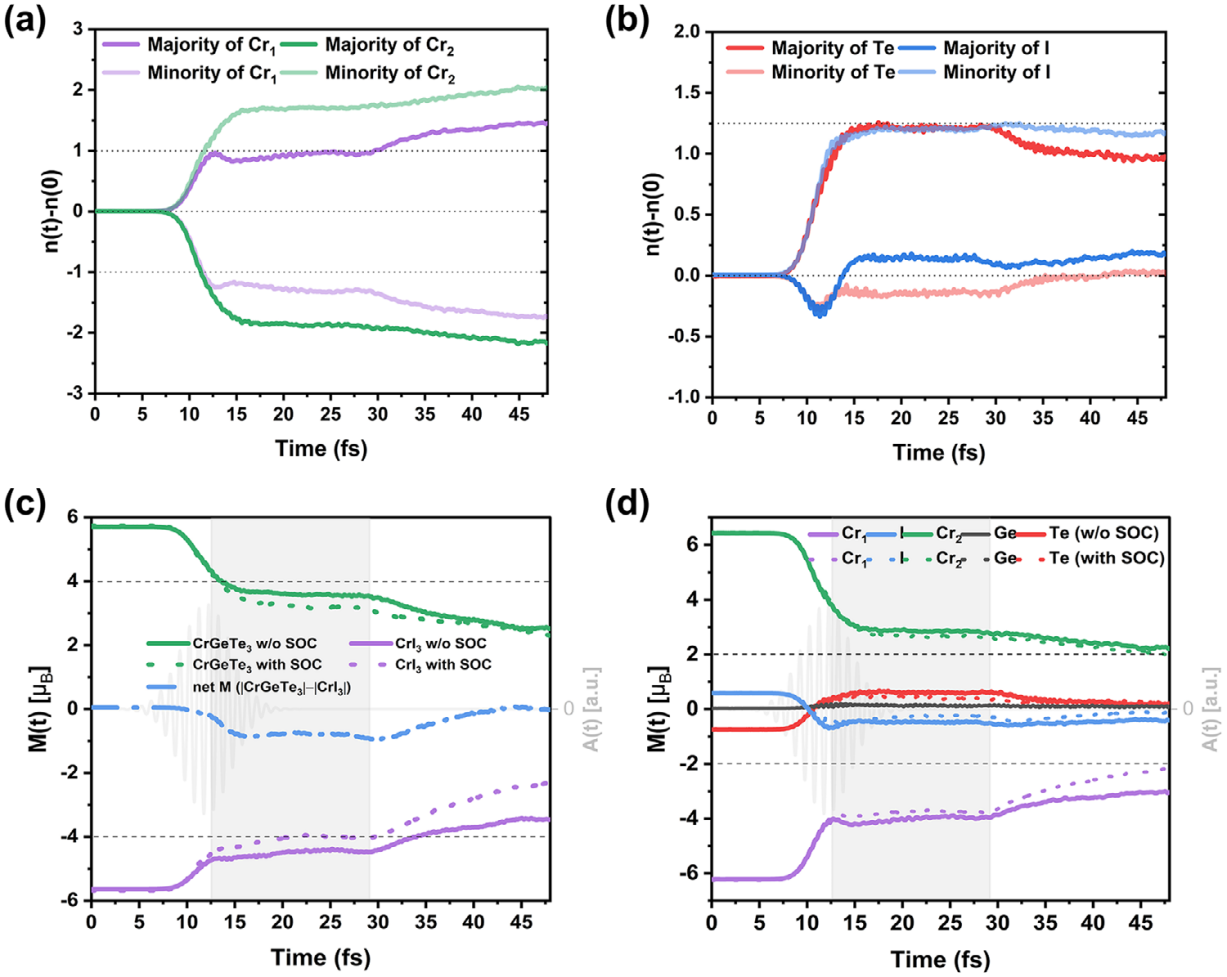
Time‐dependent occupation dynamics, and time‐dependent spin dynamics with and without SOC. a, b) Time‐dependent change in the majority and minority occupations of magnetic Cr atoms (a) and nonmagnetic I /Te atoms (b) as a function of time (in fs), defined as n(t) − n(0). c) Time‐dependent dynamics of the layer magnetic moment with and without SOC. d) Time evolution of the local magnetic moment with and without SOC, along with the vector potential A(t) of the laser pulse.

Next, we will examine the underlying physical mechanism responsible for the ultrafast demagnetization induced by the laser in the CrI_3_/CrGeTe_3_ heterostructure. To gain a deeper understanding of these phenomena, we study the time‐dependent occupation (n(t) − n(t═0)) for the different atoms (**Figure** **3**a,b). We find that the minority (majority) spin electrons of Cr_1_ atoms decrease (increase) rapidly, while the Cr_2_ atoms exhibit the opposite behavior. This process demonstrates the phenomenon of spin‐resolved charge redistribution of magnetic atoms during the demagnetization process. Meanwhile, the magnetic Cr atoms in different layers exhibit unequal spin‐electronic reconfiguration, and this process of interlayer imbalance distribution leads to asymmetric demagnetization of the magnetic atoms, which promotes the transition of the AFM state to the FiM state. Moreover, the spin‐electronic changes persist after 28 fs, suggesting that the magnetic remodeling is not only driven by the ultrafast electron redistribution but also modulated by the spin‐relaxation process on longer time scales.

As shown in Figure 3b, we show that the majority/minority spin‐resolved electron number of I and Te atoms changes in the opposite direction compared to that of magnetic Cr atoms, a signature of the OISTR mechanism. That means the laser‐induced electron transfer occurs not only within magnetic atoms, but also involves spin rearrangements in nonmagnetic atomic layers. In addition, we observe that the spin‐electron occupancy of I and Te atoms exhibits a clear asymmetric change, suggesting a charge compensation effect of the nonmagnetic atoms on the magnetic ones. In the AFM ground state, I and Te atoms form a stable electron distribution with Cr atoms through orbital hybridization, and the laser pulse disrupts this equilibrium, allowing the nonmagnetic atoms to adapt to the new magnetic environment through electron rearrangement. This charge compensation mechanism not only affects the intralayer magnetic dynamics but also further enhances the stability of the FiM states. This observation provides important time‐resolved evidence for the OISTR effect in 2D magnetic vdW materials and reveals the key role of nonmagnetic atoms in the laser‐induced ultrafast magnetic evolution.

In addition, spin‐orbit coupling (SOC) plays a crucial role in ultrafast demagnetization and interlayer spin transfer.^[^ ^31^, ^32^ ^]^ Here, we investigate the time‐evolution magnetic moment of the CrI_3_ and CrGeTe_3_ layers under SOC (Figure 3c,d). We find that SOC‐induced spin flips (SF) significantly enhance the demagnetization in the CrI_3_ layer. Compared with the demagnetization process dominated only by charge transfer (CT), the SOC further disrupts the spin conservation and flips the minority spin electrons of the CrI_3_ layer to the majority spin state, thus accelerating the magnetic moment loss of the CrI_3_ layer. Especially after 12 fs, the SOC‐induced SF effect gradually increases and becomes the main mechanism for the demagnetization of the CrI_3_ layer.^[^ ^33^ ^]^ In contrast, the CrGeTe_3_ layer is less responsive to SOC, and its demagnetization is mainly contributed by CT. The SOC does exacerbate the demagnetization of the CrGeTe_3_ layer during 15‐40 fs, but the effect is much less than that of the CrI_3_ layer. This suggests that although SOC acts throughout the system, there are significant differences in the mechanism of its action in the different layers. The CrI_3_ layer is mainly influenced by SF, while the CrGeTe_3_ layer is still dominated by CT. It is noteworthy that the SOC‐induced SF process leads to a narrowing of the interlayer magnetic moment difference after 40 fs, bringing the system back to the AFM arrangement. Due to the intensified demagnetization of the CrI_3_ layer and the relatively small change in the magnetic moments of the CrGeTe_3_ layer. This uneven demagnetization gradually diminishes, leading to a reduced interlayer magnetic moment difference and ultimately restoring the system to the AFM state. This result indicates that the SOC‐induced SF process in CrI_3_/CrGeTe_3_ heterostructures enhances the local demagnetization, and drives the ultrafast magnetic phase transition of the system by changing the interlayer magnetic moment distribution.

### 2.3 Spin Relaxation Dynamics After Laser Pulse

**Figure 4:**
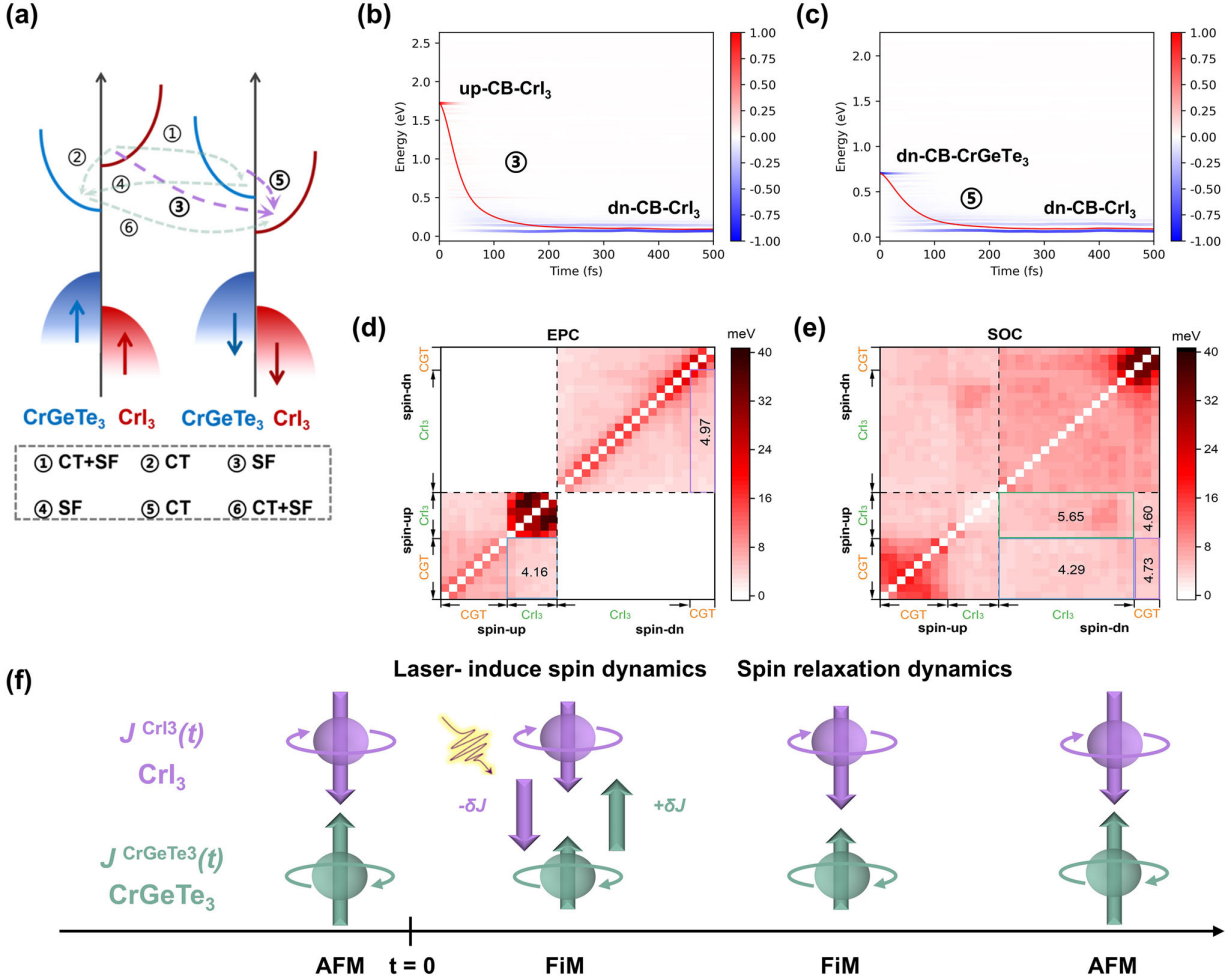
a) Schematic of spin‐electron relaxation pathways in the CrI_3_/CrGeTe_3_ heterostructure. The bold dashed arrows indicate the most probable dynamics relaxation pathway: spin‐flip (SF) and charge transfer (CT). The arrows pointing upward (downward) correspond to spin‐up (spin‐down) channels, respectively. b,c) Energy relaxation of excited spin‐up electrons in the CrI_3_ layer and the spin‐down electrons in the CrGeTe_3_ layer. The color map illustrates the orbital localization. d)The EPC and e) SOC between electronic states in the CrI_3_/CrGeTe_3_ heterostructure based on the spin‐diabatic representation. The average NAC values between the key states are listed. f) Schematic diagram of the time evolution of laser‐induced magnetic state transition.

As we mentioned, the ultrafast spin dynamics of the CrI_3_/CrGeTe_3_ heterostructure exhibit distinct layer‐dependent and nonequilibrium properties. However, upon the disappearance of the laser pulse, the interlayer magnetic moment reconstruction involves spin relaxation on a longer timescale. The excited spin electron undergoes relaxation from the higher conduction band (CB) to the lower CB, potentially influencing the magnetic order of the CrI_3_/CrGeTe_3_ heterostructure (**Figure** **4**a). To examine the evaluation of magnetic order after the laser disappears, we calculate the time‐dependent energy relaxation of the excited electron in the CrI_3_/CrGeTe_3_ system using ab initio NAMD simulations, as shown in Figure 4b,c. Spin electron relaxation processes can be divided into six potential pathways that involve spin‐flip (SF) and charge transfer (CT). We observe that the excited electron of the CrI_3_ layer relaxes from the spin‐up channel to the spin‐down channel in the CrI_3_ layer, forming an intralayer SF. In this case, the magnetic moment of the CrI_3_ layer will recover due to the decrease of electron population between the majority state Δn_↑_ and minority state Δn_↓._ Meanwhile, the excited electron in the CrGeTe_3_ layer undergoes an interlayer CT from the spin‐down channel of CrGeTe_3_ to the spin‐down channel of CrI_3_, leading to the magnetic moment recovery of CrGeTe_3_ and simultaneously further increasing the spin‐down electron of CrI_3_. Note that the intralayer SF and interlayer CT processes occur within time scales of 40 fs and 53 fs, respectively, which are fitted by an exponential function. Such asymmetric electron relaxation will lead to an inequivalent evolution in the magnetic moment, thus inducing a FiM state in the CrI_3_/CrGeTe_3_ heterostructure on a tens of femtoseconds time scale.

To elucidate the mechanism of spin relaxation behavior in the CrI_3_/CrGeTe_3_ system, we perform the calculation of nonadiabatic coupling (NAC) between key states based on spin‐diabatic representation.^[^ ^29^, ^34^ ^]^ This framework enables one to distinguish the contribution of the electron‐phonon coupling (EPC) component and the SOC component (Figure 4d,e). In particular, the EPC component only contributes to the contiguous orbitals with the same spin states. However, the SOC component enables coupling not only between same‐spin states but also between opposite‐spin states. The larger the NAC is, the faster the spin electron relaxes. We note that the SOC component (5.65 meV) between the spin‐down and spin‐up states of the CrI_3_ layer is larger than the EPC component (4.16 meV) of spin‐up states between the two layers and the SOC component (4.60 meV) between spin‐up states of the CrI_3_ layer and spin‐down states of the CrGeTe_3_ layer. Meanwhile, the EPC component (4.97 meV) of spin‐down states between the two layers is larger than the SOC component (4.73 meV) between spin‐up and spin‐down states in the CrGeTe_3_ layer. Such results explain why the intralayer SF (process ③) prefers to occur for the spin‐up electron of CrI_3_ and interlayer CT (process ⑤) for the spin‐down electron of CrGeTe_3_. Besides, the lattice thermal fluctuation will alter the position of ions during the electron relaxation process, inducing phonon modes participating in the electron relaxation via electron‐phonon interaction. Figure S8 (Supporting Information) depicts the Fourier transform spectra of the band edge states of the CrI_3_ and CrGeTe_3_ layers. The main vibration peaks appear at 100.39, 133.09 and 121.47 cm^−1^, respectively, corresponding to the spin‐up state of the CrGeTe_3_ layer, the spin‐up state of the CrI_3_ layer, as well as the spin‐down state of two layers. That means the lattice fluctuation causes the spin‐down state of the CrI_3_ layer instead of the spin‐up state of the CrGeTe_3_ layer to primarily participate in the electron relaxation process due to the higher vibration frequency for the former, favoring the generation of the intralayer SF and interlayer CT paths.

Based on the rt‐TDDFT and NAMD results, we provide a comprehensive physical picture of the laser‐induced magnetization transition (Figure 4f). During the laser excitation, the asymmetric spin dynamics in the CrI_3_/CrGeTe_3_ heterostructure disrupt the interlayer spin arrangement, which prompts the transition of the system from the AFM state to the FiM state. When the laser disappears, the intralayer SF in the CrI_3_ layer and interlayer CT between the CrI_3_ and CrGeTe_3_ layers occur within tens of femtoseconds. This results in an asymmetric magnetic moment recovery in two layers because of their different electron relaxation times (40 fs v.s. 53 fs). Such an inequivalent electron relaxation process breaks the interlayer AFM symmetry, thus maintaining the ultrafast FiM state before recovering to an AFM state.

## 3 Conclusion 

In summary, this study employs rt‐TDDFT and NAMD to explore the ultrafast laser‐induced magnetic phase transition in the CrI_3_/CrGeTe_3_ van der Waals heterostructure. Our findings reveal that laser excitation triggers a nonequilibrium spin dynamics, leading to an ultrafast transition from the antiferromagnetic state to the ferrimagnetic state. This transition is driven by the asymmetric demagnetization of magnetic atoms and the compensation effect of nonmagnetic atomic magnetic moments, with a crucial role played by the OISTR mechanism. In spin relaxation processes, we uncover distinct relaxation mechanisms in the CrI_3_ and CrGeTe_3_ layers. The CrI_3_ layer is dominated by spin‐flip processes mediated by SOC, resulting in rapid spin relaxation, whereas the CrGeTe_3_ layer primarily exhibits charge‐transfer driven relaxation with a weaker SOC effect. This asymmetric interlayer relaxation increases the lifetime of the FiM state. Our study elucidates the fundamental mechanism of laser‐driven magnetic phase transitions and spin relaxation dynamics process in CrI_3_/CrGeTe_3_ heterostructures, providing theoretical insights for optically controlled magnetic storage and ultrafast spintronic devices while advancing the understanding of nonequilibrium spin dynamics in 2D magnetic materials.

## 4 Computational Methods

Ground state properties were carried out with the Vienna Ab initio Simulation Package (VASP).^[^ ^35^, ^36^ ^]^ The exchange‐correlation effects was treated using the PBE functional within the generalized gradient approximation (GGA).^[^ ^37^ ^]^ The PAW method was employed to represent the electron‐ion interaction.^[^ ^38^ ^]^ A plane‐wave basis with an energy cutoff of 500 eV and a 6×6×1 Monkhorst‐Pack k‐point grid was applied for both geometry optimization and electronic structure computations. The lattice constants and atomic coordinates were relaxed until the atomic forces were below 0.01 eV Å^−1^, and an electron relaxation convergence threshold set to 10^−5^ eV. To better describe the Cr‐d orbital, a Hubbard U correction of 3 eV was applied.^[^ ^39^ ^]^ The vdW weak interaction was considered using the Grimme DFT‐D3. A 15Å vacuum layer was introduced along the out‐of‐plane direction to suppress artificial coupling between periodic images.

Laser‐induced spin dynamics were simulated using the ELK code^[^ ^40^ ^]^ with the fully noncollinear version of rt‐TDDFT. Simulations employed 6×6×1 k‐point mesh, a smearing width of 0.027 eV, and a time step of Δtɐ0.2 a.u., within the adiabatic local spin density approximation (ALSDA) framework, with Hubbard U (ALSDA+U), with U═3 eV for Cr atoms.

The spin‐electron relaxation dynamics simulations were implemented with the Hefei‐NAMD code (Hefei‐NAMD_SOC version).^[^ ^41^, ^42^ ^]^ This relaxation process was described by the quantum‐classical fewest switches surface hopping (FSSH) method. After the geometry structures were optimized using VASP, the relaxed structures were thermalized at 300 K over 2 ps through repeated velocity rescaling. Subsequently, a 3 ps ab initio molecular dynamics (AIMD) trajectory was generated in the microcanonical ensemble with a time step of 1 fs. Two hundred initial configurations were randomly extracted from the first 1 ps AIMD trajectory, and 20000 NAMD trajectories were generated for each chosen structure. The NAC was evaluated in the spin‐diabatic representation, which enables a clear contribution of EPC and SOC components.^[^ ^42^, ^43^ ^]^

## Conflict of Interest

The authors declare no conflict of interest.

## Supporting information



Supporting Information

## Data Availability

The data that support the findings of this study are available in the main text and supplementary material of this article.
