# Supplementary material for: Laser‐Induced Ultrafast Magnetic Phase Transition in 2D Van Der Waals Antiferromagnetic Heterostructures
Source: Adv Sci (Weinh). 2026 Jan 5:e15533. Online ahead of print. doi: 10.1002/advs.202515533 (PMC13325505; doi:10.1002/advs.202515533)
Supplement: Supplementary file 1 — Supporting Information [file ADVS-9999-e15533-s001.docx]

Supporting Information

Laser-Induced Ultrafast Magnetic Phase Transition in 2D van der Waals Antiferromagnet Heterostructures

Yang Wu, Fulu Zheng, San-Dong Guo, Thomas Frauenheim*, Zhaobo Zhou*, Junjie He*

**Supplementary Figures**


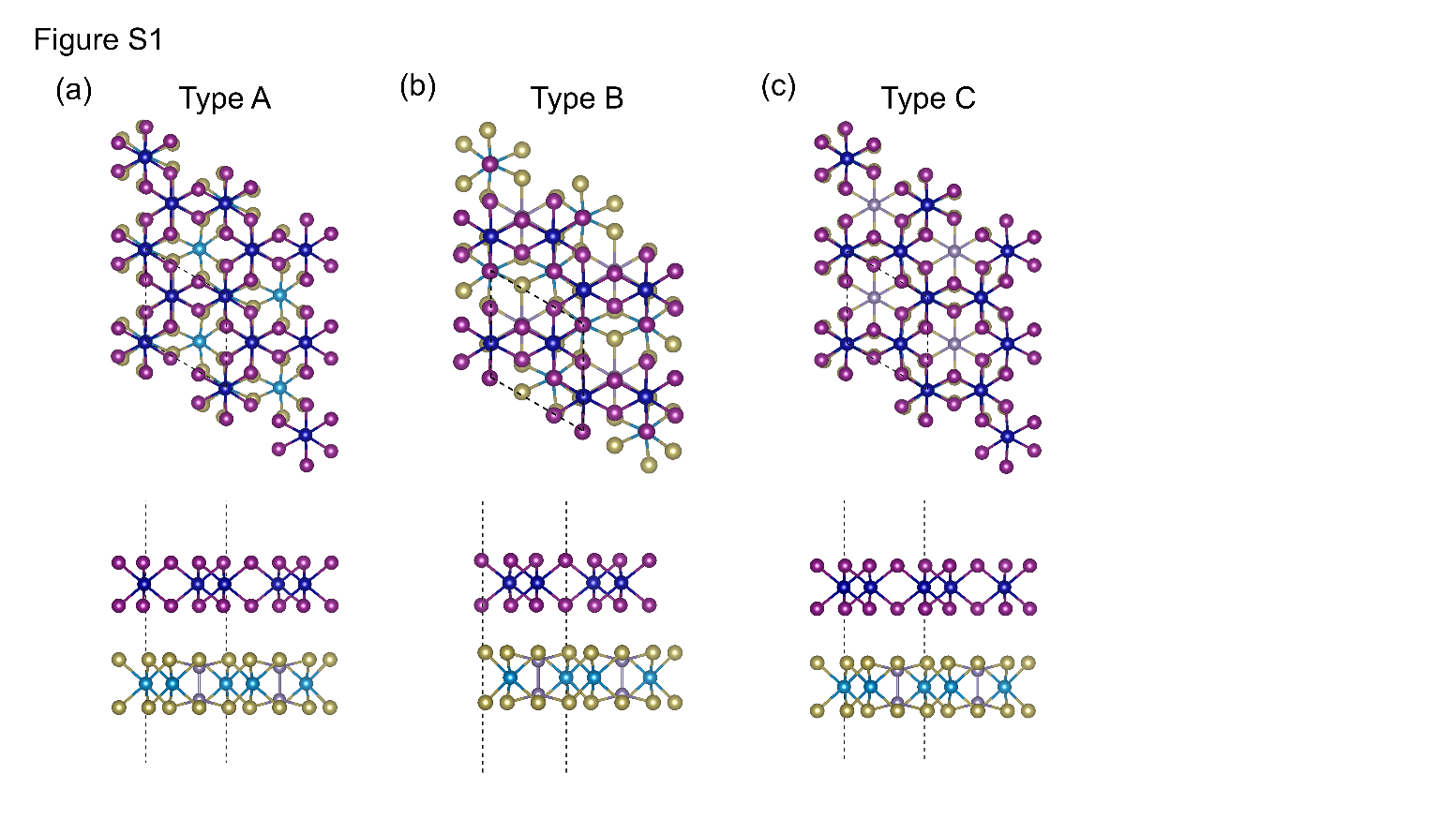


**Figure S1.** (a-c) Top and side views of different stacking configurations for CrI_3_/CrGeTe_3_ heterostructures at the interface.


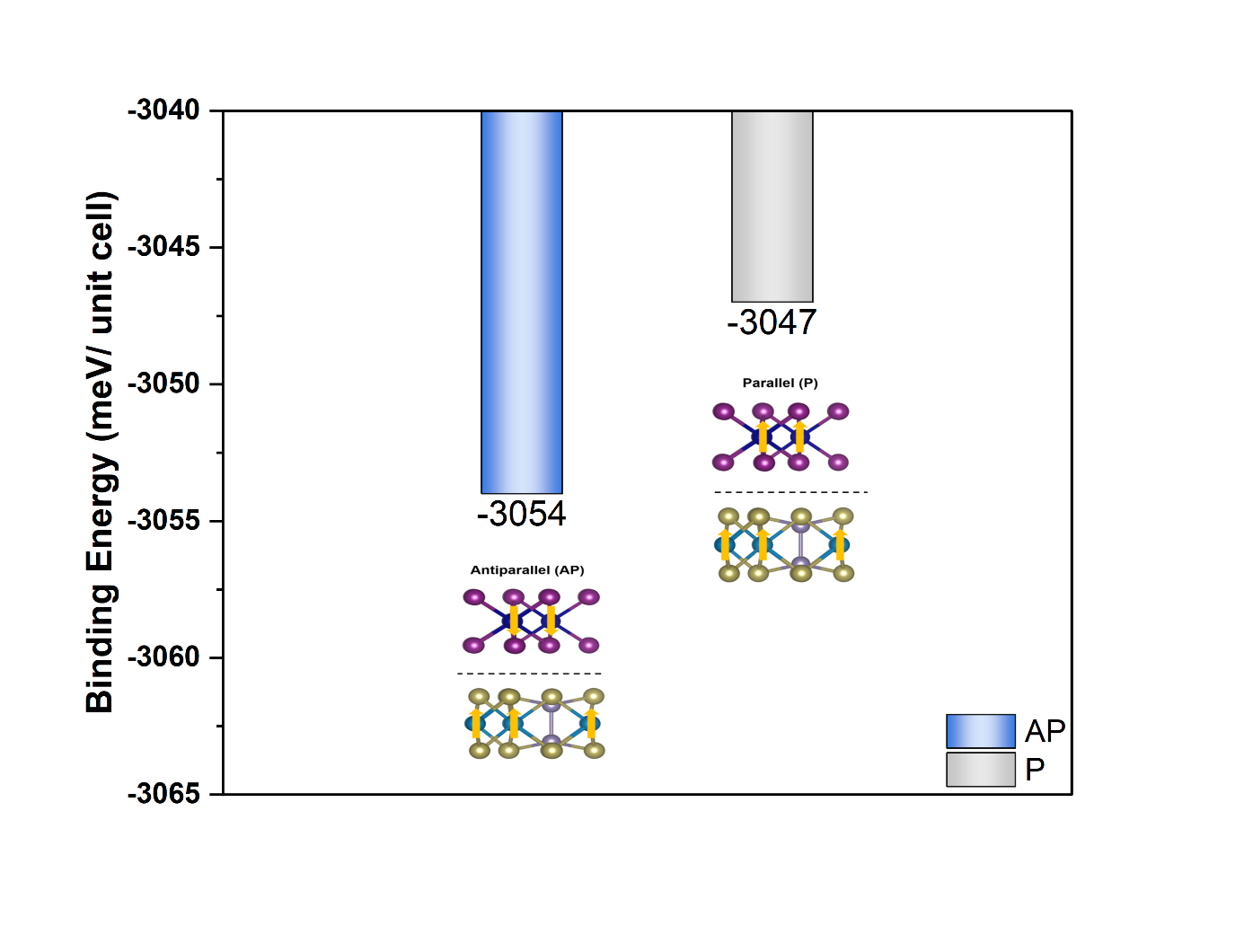


**Figure S2.** Comparison of the binding energies of the AP and P configurations in the CrI_3_/CrGeTe_3_ heterostructure.


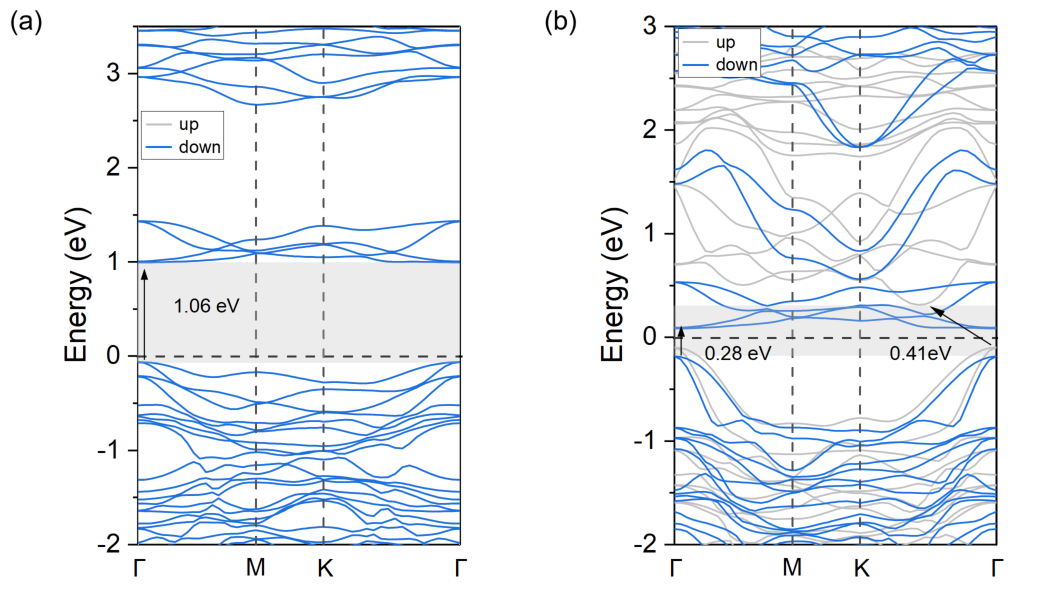


**Figure S3.** (a, b) Band structures of the AB-stacked bilayer CrI_3_ and CrI_3_/CrGeTe_3_ heterostructures.


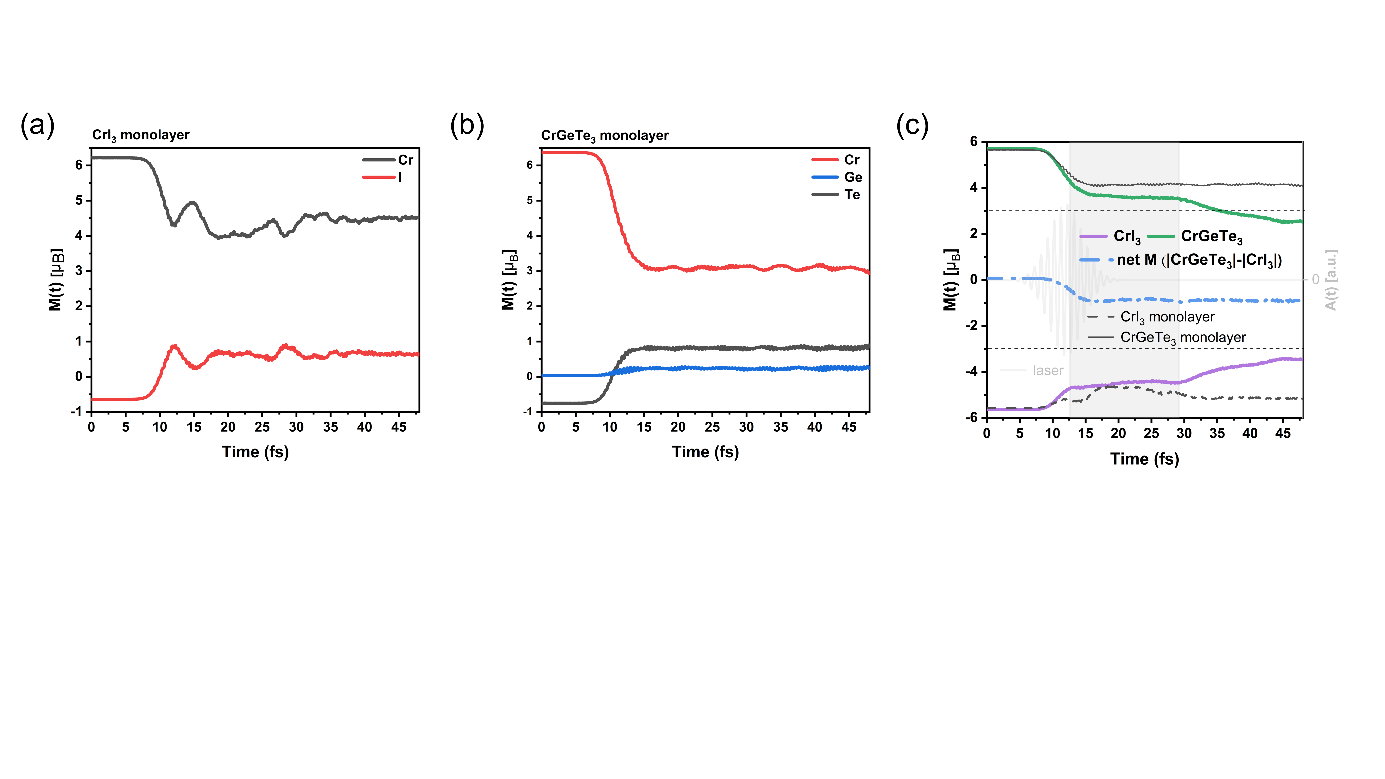


**Figure S4.** (a, b) Time evolution of the local magnetic moment of the CrI_3_ and CrGeTe_3_ monolayer (c) Time-dependent dynamics of the layer magnetic moments for the CrI_3_ monolayer, CrGeTe_3_ monolayer, and the CrI_3_/CrGeTe_3_ heterostructure, along with the vector potential A(t) corresponding to the laser pulse. For a direct comparison with the heterostructure, the demagnetization curve of the CrI_3_ monolayer is plotted with inverted values.


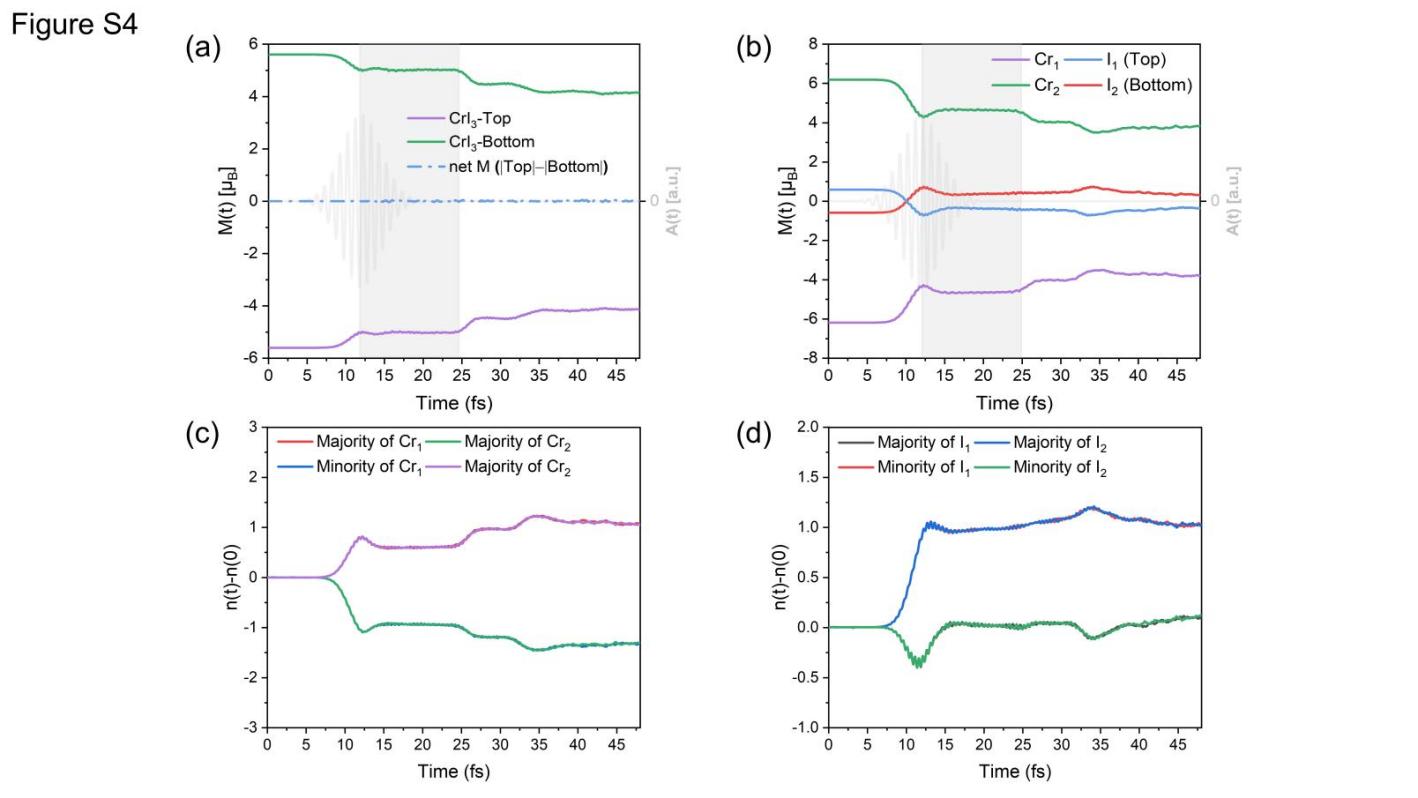


**Figure S5.** (a) Time-dependent dynamics of the layer magnetic moment for CrI_3_-Top (violet) and CrI_3_-Bottom (green) in the AB-stacked bilayer CrI_3_. (b) Time evolution of the local magnetic moment. The vector potential A(t) of the laser pulse is also shown. (c, d) Time-dependent change of majority and minority occupations as a function of time (in fs) of magnetic Cr atoms and nonmagnetic I /Te atoms (right), which is defined as n(t) − n(0). The calculations on bilayer CrI_3_ reveal symmetric demagnetization dynamics, where the two magnetic layers experience nearly identical spin moment reduction under laser excitation. This behavior is consistent with the structural and spin occupations equivalence of the two layers, preserving the net-zero magnetization throughout the excitation process.


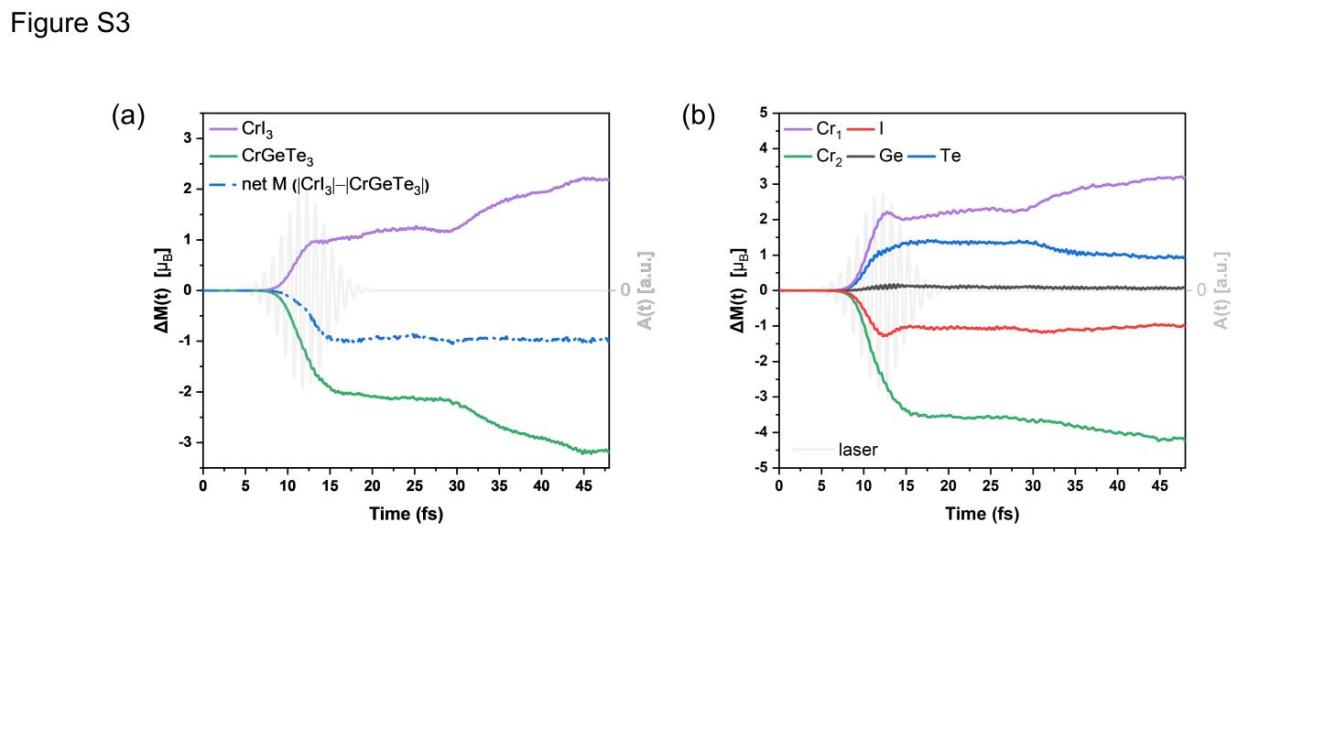


**Figure S6.** Time-dependent changes in the magnetic moment, *ΔM(t)=M(t) − M(t=0)*, for (a) different layers in CrI_3_/CrGeTe_3_ heterostructures. (b) Element-resolved magnetic moment changes for Cr, I, Ge, and Te as a function of time (in femtoseconds).


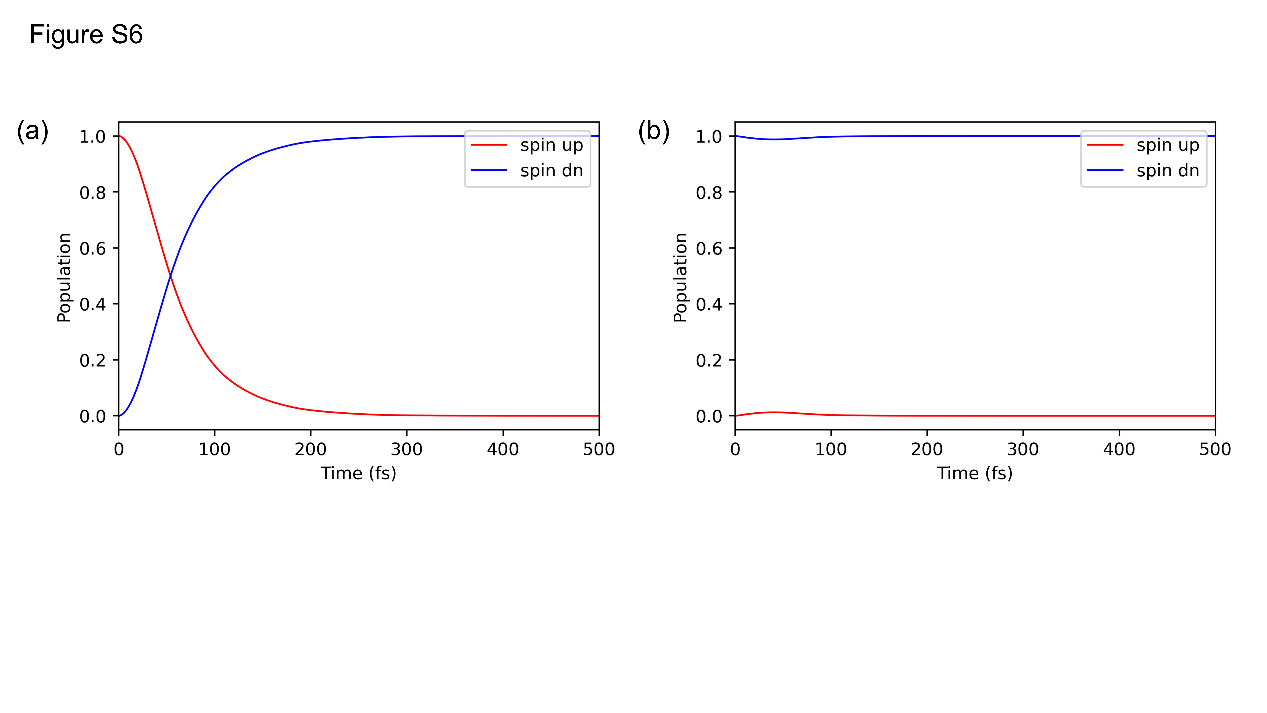


**Figure S7.** (a, b) Spin population distributions of relaxed spin-polarized electrons in the CrI_3_/CrGeTe_3_ heterostructure, corresponding to processes ③ and ⑤, respectively. The red and blue lines represent the probability that spin electrons localize in the spin-up and spin-down channels, respectively.


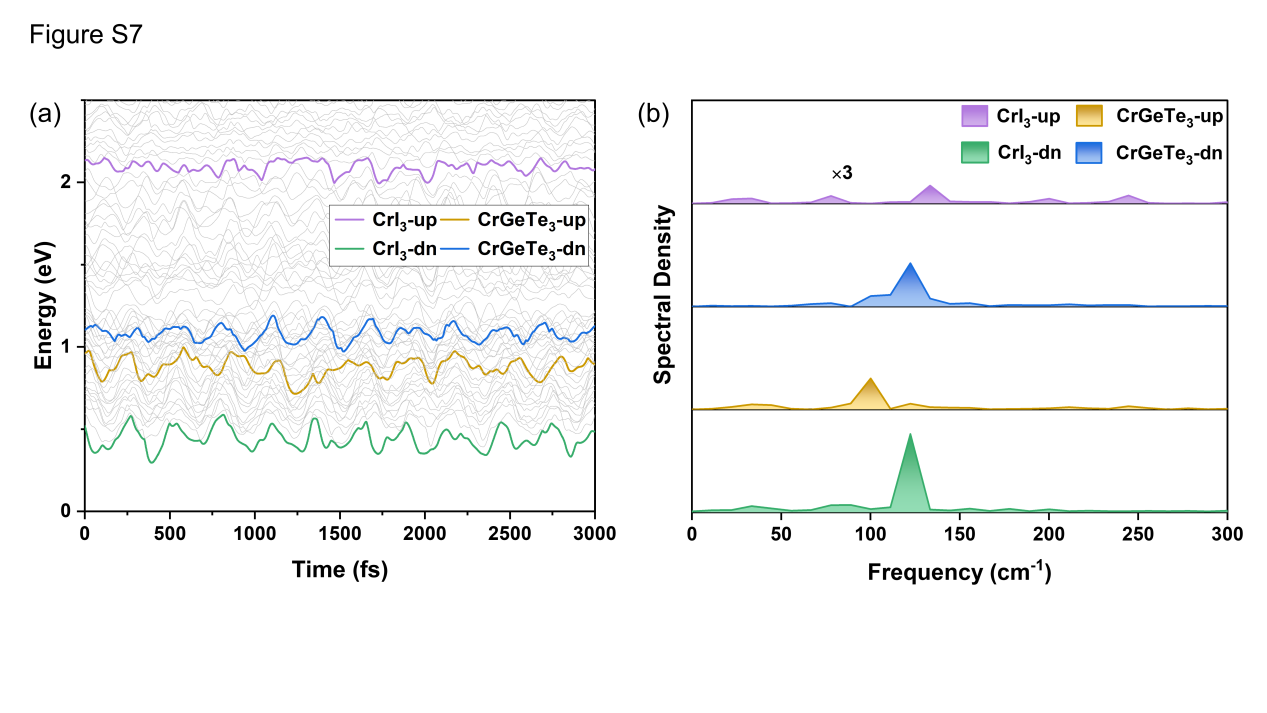


**Figure S8.** (a) Energy evolution of electronic states of CB at the Γ point and (b)Fourier transforms spectral densities of corresponding key states.

**Figure S9.** Fourier transform spectral densities of each state in the CrI_3_/CrGeTe_3_ heterostructure.

**Supplementary Table**

**Table S1.** The Binding energy of the CrI_3_/CrGeTe_3_ heterostructures with the type A, B and C stacking configurations. The d is the interlayer distance (Å) between CrI_3_ and CrGeTe_3_ layers.

| System | Type A | **Type B** | Type C |
| --- | --- | --- | --- |
| Binding energy (eV) | -2.832 | **-3.054** | -2.809 |
| d (Å) | 3.971 | **3.381** | 3.988 |
